# Progress in Hyaluronan-Based Nanoencapsulation Systems for Smart Drug Release and Medical Applications

**DOI:** 10.3390/molecules30193883

**Published:** 2025-09-25

**Authors:** Katarína Valachová, Mohamed E. Hassan, Tamer M. Tamer, Ladislav Šoltés

**Affiliations:** 1 Centre of Experimental Medicine of Slovak Academy of Sciences, Dubravska cesta 9, 84104 Bratislava, Slovakia; 2 Centre of Scientific Excellence-Group of Advanced Materials and Nanotechnology, Chemistry of Natural and Microbial Products Department, National Research Centre, El Behouth Street, Cairo 12622, Egypt; mohassan81@gmail.com; 3 Polymer Materials Research Department, Advanced Technologies and New Materials Research, Institute (ATNMRI), City of Scientific Research and Technological Applications (SRTA-City), New Borg El-Arab City, Alexandria 21934, Egypt

**Keywords:** encapsulation, hyaluronan, hydrogels, medicine, nanoparticles, polysaccharides

## Abstract

Hyaluronan (HA), a high-molecular-weight polysaccharide naturally found in vertebrate tissues such as skin, joints, and the vitreous body, plays a critical role in various biological processes. Its functionality is highly dependent on molecular weight, with high-molecular-weight HA exhibiting anti-inflammatory and immunosuppressive effects, while low-molecular-weight HA promotes inflammation, immunostimulation, and angiogenesis. Due to its biocompatibility, biodegradability, and tunable properties, HA has gained increasing attention in biomedical applications. This review summarizes recent advances in the encapsulation of HA with other polymers and therapeutic agents in nanosystems, particularly hydrogels and nanoparticles. HA-based formulations demonstrate improved therapeutic outcomes, including drug release sustained up to 7 days, wound closure rates exceeding 90% in animal models, particle size in the range of 50–300 nm, and enhanced bioavailability of encapsulated drugs by 2–3 fold compared with free drugs. Such properties have shown promise in enhancing therapeutic efficacy and targeted drug delivery in the treatment of skin wound healing, diabetes, osteoarthritis, rheumatoid arthritis, and ophthalmic diseases. The review emphasizes how HA’s modifications and composite systems optimize drug release profiles and biological interactions, thereby contributing to the development of next-generation biomedical therapies.

## 1. Introduction

Encapsulation is a process in which a core material is entrapped in polymeric coatings [1,2,3]. The core material, defined as the specific substance to be coated, may exist in a liquid, solid, or gaseous state. The liquid core can encompass either dispersed or dissolved materials, while the solid core may consist of active constituents, excipients, stabilizers, release-rate retardants, diluents or accelerators. The release of the core material from the nano/microcapsules can be engineered to respond to physiological changes in the human body, such as variations in pH, enzyme activity, and temperature. This engineering provides control over the rate at which the active ingredient is released and absorbed by the body, thereby maximizing therapeutic efficacy and minimizing adverse effects [3,4]. Based on the dispersion of the core material, encapsulated particles can be categorized into 1) matrix systems, wherein an active component or core is physically and uniformly dispersed, and 2) vesicular systems, characterized by the confinement of the core material within a cavity encircled by a polymer membrane (capsules). The coating material, also referred to as a shell, must form a cohesive film with the core material, exhibiting chemical compatibility and non-reactivity with the core material, and providing the desired coating properties, including strength, flexibility, impermeability, optical and functional characteristics, and stability [3]. Commonly utilized coating polymers include 1) natural polymers such as polysaccharides (e.g., alginate, chitosan, agarose, hyaluronic acid, dextran), 2) proteins (e.g., albumin, gelatine, zein), 3) synthetic polymers (e.g., poly-ε-caprolactone, polyethylene glycol, polylactic-co-glycolic acid), and 4) other coating compositions that may involve plasticizers, coloring agents (in pharmaceutical and food applications), lipids, waxes, resins, and release rate modifiers [1,2,3,5,6,7,8,9].

**Figure 1:**
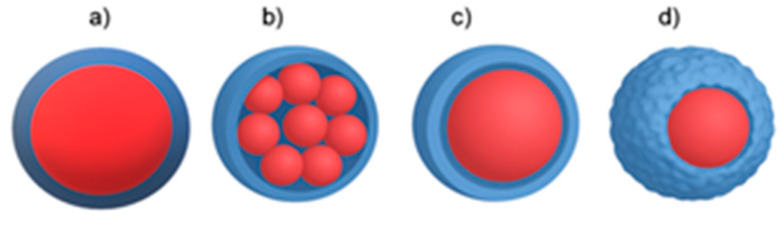
Common microcapsule morphologies: (**a**) continuous core–shell capsule, with a single core surrounded by a uniform shell; (**b**) polycore capsule, containing multiple discrete cores within one shell; (**c**) multilayered core–shell capsule, in which a continuous core is encapsulated by more than one shell layer; (**d**) matrix-type capsule, where the active material is homogeneously dispersed throughout the polymer matrix (adapted from [11]).

Encapsulation techniques can be categorized as follows:

1. Physical methods: Spray drying, spray cooling, air suspension, envelope-combination, extrusion, supercritical solution processing, porous centrifugal, electrostatic binding, solvent evaporation, and rotary separation.
2. Chemical methods: This category encompasses interfacial polymerization, in situ polymerization, and piercing-solidifying.
3. Physical–chemical methods: Simple and complex coacervation, phase separation, drying bath, powder bed grinding, melting-dispersion condensation, and capsule-core exchange [7,10]. Figure 1 illustrates the various modes of how a core material (depicted in red) can be encapsulated into coating materials.

Based on their diameter, capsules can be classified into three categories: macrocapsules (>5000 µm), microcapsules (0.2 to 5000 µm) and nanocapsules (<0.2 µm) [12].

The primary factors determining successful encapsulation include the complete formation of the shell, the absence of leakage, and the prevention of impurities that could adversely affect the properties of phase change material. Additionally, it is essential to consider the encapsulation ratio and the encapsulation efficiency [13].

Advantages of encapsulation are: 1) to protect core material from UV radiation, oxidation, heat, acids, bases, 2) to prevent degradation reactions by improving shelf life, 3) to disguise bitter taste and odors, 4) to control hygroscopicity, 5) to handle liquids as solids, 6) to enhance flow ability, solubility and permeability [14].

Despite the significant benefits of encapsulation, several potential drawbacks warrant consideration: 1) expensive production processes, particularly at larger scales, 2) reduced shelf life for hygroscopic agents encapsulated within the particles due to potential moisture absorption, and 3) inconsistencies in the coating can affect the release of encapsulated materials, potentially leading to variability in performance [15,16,17,18,19].

Since the 1940s, microencapsulation has emerged as a compelling method due to its various applications [8], including the textile industry, agriculture (encapsulation of pesticides, biocides, and agrochemicals), fragrances, phase-change materials, cosmetics, and incorporation of coatings (antimicrobial, antifungal). In medicine, microencapsulation is used in the subcutaneous and intramuscular delivery of analgesics, the arterial and intratumoral delivery of anticancer agents, wound healing, and the treatment of cardiovascular and renal diseases, as well as anemia. Moreover, encapsulated drugs have been used in hormone therapy, gastrointestinal disorders, diabetes, pulmonary diseases, periodontitis, hypertension, and self-healing materials [2,6,8,20,21]. Currently, the development of bifunctional micro- or nano-capsules, doped high-performance materials, and copolymer encapsulation is particularly promising when compared with monofunctional capsules comprising pure polymer shells [13]. Encouraging clinical findings have shown the potential beneficial applications of microencapsulation for islet transplantation as well as for cardiac, neuronal, and hepatic repair [17,18].

The aim is to summarize the progress in hyaluronan-based encapsulation of natural and synthetic compounds, cells, and growth factors in medicine, particularly in skin wound healing, diabetes, osteoarthritis, rheumatoid arthritis and ophthalmology in the years 2020-2025.

## 2. Encapsulated Materials

### 2.1. Stem Cells

Stem cell therapy and the application of biomaterials for drug delivery as innovative therapeutic approaches have recently garnered significant attention in clinical practice. These methodologies facilitate shorter hospitalization, more precise and timely diagnostics, and less invasive and faster treatment. Stem cell therapy has been performed to treat musculoskeletal, neurological, and cardiovascular diseases and osteoarthritis (OA).

Stem cells possess the unique capability of self-renewal and can differentiate into two primary categories: 1) pluripotent stem cells, such as embryonic stem cells and induced pluripotent stem cells, and 2) multipotent stem cells such as hemopoietic stem cells and adult stem cells. The process of cell encapsulation involves the immobilization of cells, allowing for synergistic intracellular interactions while protecting them from being washed out or damaged by surrounding shear forces. Cell encapsulation cells are safeguarded against hydrodynamic pressure and aggregation while enabling the functional diffusion of nutrients, growth factors, and gases through the microcapsule matrix. Suitable materials for cell encapsulation should mimic the extracellular matrix (ECM) and should be processed under conditions compatible with the presence of cells [15]. Optimal materials for cell encapsulation should be nontumorigenic, well-defined, easily accessible, and reproducible. The encapsulating material is necessary to protect cells from the host’s immune system, provide nutrient and waste exchange through appropriate pore sizes, and prevent interference with host cell homeostasis. Mechanical properties of material are critical for cell protection and controlled release of encapsulated molecules [22]. However, challenges persist in the field of encapsulation, including maintaining the viability of encapsulated cells, managing the rate of polymer membrane degradation, addressing pericapsular fibrosis formation, and minimizing the immunogenicity of polymers [15,16,17,18].

### 2.2. Hydrogels and Nanoparticles

**Figure 2:**
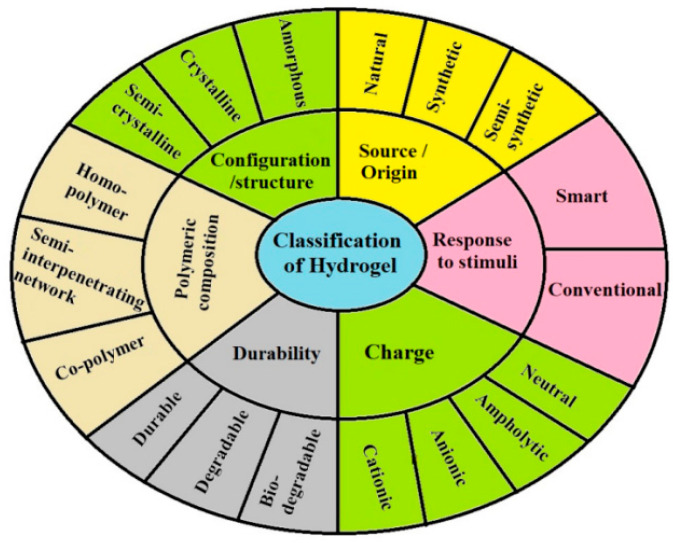
Classification of hydrogels (adapted from Ali et al. [29]).

The nonspecific distribution and uncontrolled drug release, which are inherent characteristics of conventional drug delivery systems, have encouraged researchers to develop smart drug delivery systems. These advanced systems offer the advantage of delivering drug molecules selectively to the desired site in the body whilst reducing dosage repetition and minimizing side effects. One of these systems is hydrogels [23], which are materials composed of cross-linked polymers that form soft and highly porous 3D networks [15]. The major properties of hydrogels include swelling, wettability, stiffness and strength, biocompatibility, biodegradability, mechanical, rheological, and biological properties such as cell adherence, migration, proliferation, differentiation, viability, angiogenesis, and vascularization [23,24,25,26]. Moreover, they are used in biological research and tissue engineering for the repair of damaged tissues, as they imitate the ECM with intricate structural and functional characteristics [23,24,25,26,27]. However, hydrogels still face several challenges, such as cell viability, growth factor burst release, or low oxygen content [28]. Hydrogels can be classified according to source, composition of a polymer, structure, response to stimuli, durability, and charge, as depicted in Figure 2.

Recently, the application of nanotechnology in drug delivery systems has undergone significant development, resulting in transformative effects within the biomedical field. Currently developed nanoscale complexes consist of a nanovehicle (a carrier such as nanoparticles (NPs)) and an encapsulated material. The drug is usually confined within a membrane or a matrix and can also be absorbed, dissolved, or dispersed from the system. Nanoparticles can be used to provide targeted delivery of a drug to the specific site and thus enhance the uptake of poorly soluble drugs and their bioavailability [28,30]. They can encapsulate one or multiple agents, including small molecules, peptides, proteins, and nucleic acids [31]. Nanostructures are adapted to protect drugs from hydrolytic degradation, which often occurs in aqueous biological environments (e.g., blood plasma, gastrointestinal tract). Encapsulation of drugs within nanocarriers protects them from direct contact with water or hydrolytic enzymes [32] and enzymatic degradation, e.g., esterases and proteases, respectively. Nanostructures act as a physical barrier, blocking enzymes from accessing the drug. In addition, surface modification can also repel enzymatic activity [33,34]. Nanostructures also restrain drugs from first-pass metabolism and increase the blood residence time. The nano size allows them to penetrate through the tissues efficiently and may also pass biological barriers [28,30]. Many orally administered drugs undergo extensive metabolism in the liver (first-pass effect), reducing systemic bioavailability. Nanocarriers can bypass this drawback by a) lymphatic uptake using lipid-based nanostructures (like nanoemulsions or liposomes), which can enhance absorption into intestinal lymphatics, thereby directly entering systemic circulation and avoiding liver metabolism [35], and b) by controlled release and mucoadhesion. In this case, NPs prolong residence in the gut, allowing gradual absorption at sites with less metabolic activity [35], e.g., self-nanoemulsifying drug delivery systems, which can improve lymphatic transport of lipophilic drugs and reduce hepatic metabolism [36]. Formulation methods are as follows: 1) nanoprecipitation, which is appropriate for engineering polymeric NPs [37], 2) high-pressure homogenization, which can be applied for engineering nanostructured lipid carriers [38], 3) thin-film hydration, which allows for encapsulating hydrophilic or hydrophobic drugs into liposomes [39], and 4) emulsification–solvent evaporation, which is used for engineering of polymeric or lipid NPs [37].

**Figure 3:**
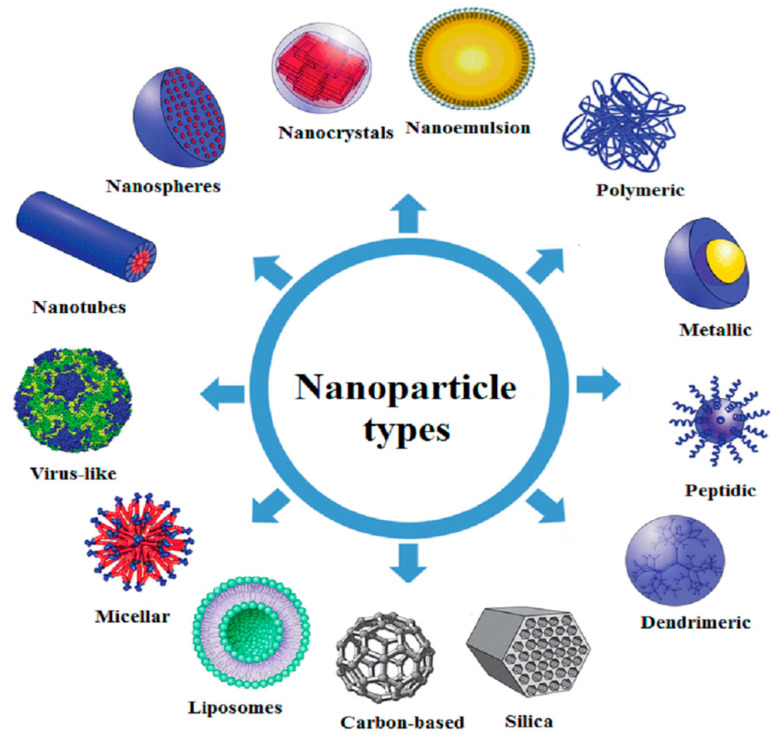
Classification of NPs (adapted from Nadaroglu et al. [42]).

Besides drug delivery, currently, there is a growing interest in using NPs for microfluidics, biosensors, microarrays, and tissue micro-engineering for the specialized treatment of diseases [40]. Moreover, NPs provide a superior cellular environment, enabling the generation of cutting-edge synthetic tissues due to their special qualities, such as stimuli sensitivity and reactivity [23,24,25,26,27]. However, despite their advantages, nano-carriers face several challenges, including complex design, time-consuming process, high-cost products, low product yield, poor cellular internalization, and therapeutic efficacy if not synthesized precisely [41]. Figure 3 displays various types of NPs.

### 2.3. Nanoparticles–Hydrogel Structures

Pure hydrogels frequently exhibit limited mechanical performance, leading to suboptimal strength, compressibility, and elasticity characteristics. Further, the inherently hydrophilic nature of hydrogels poses challenges in achieving high loading and sustained release of certain drugs due to the inherently hydrophilic nature of hydrogels. For this reason, the incorporation of NPs into hydrogels yields novel superstructures that have become increasingly popular in biomedical research. They are generally composed of hydrated, crosslinked polymeric networks incorporated with NPs. Encapsulation of drug-loaded NPs within a hydrogel matrix is also a strategy that has been used to prevent rapid drug release. For localized administration to an affected area, NP–hydrogels provide a promising approach for the delivery of both hydrophobic and hydrophilic drugs [31].

## 3. Hyaluronic Acid

Currently, biopolymers used in medicine are, e.g., silk–agarose, collagen, fibroin, chitosan, and hyaluronic acid (hyaluronan, HA) [43]. This review focuses on the applications of HA, since it is a linear, natural, and unbranched polysaccharide found in human tissues. It is composed of repeating disaccharide units of d-glucuronic acid and *N*-acetyl-d-glucosamine, connected by β-1,4 and β-1,3 glycosidic bonds [44,45,46]. Hyaluronan has biocompatible, non-toxic, biodegradable, and nonimmunogenic properties [47,48,49]. Hyaluronan hydrogels facilitate the encapsulation and controlled release of drugs, growth factors, or cells [39]. Additionally, HA possesses antibacterial properties and can act as a stimulus-responsive agent. However, HA has a low level of cell adherence and rapidly disappears after injection [23]. For these reasons, it is necessary to chemically modify HA at carbonyl, hydroxyl, or *N*-acetyl groups that enable cross-linking between polymer chains and highly tunable scaffold formation, thereby improving their biophysical tunability, including mechanical properties, while maintaining their biological activity. Besides hydrogels, HA-based microspheres and composite hydrogel systems can be fabricated [25]. Upon exposure to physiological stimuli such as pH and temperature changes or enzymatic activity, the hydrogel matrix undergoes structural modifications that trigger controlled drug release. This stimuli-responsive behavior ensures sustained and site-specific delivery, thereby enhancing bioavailability, minimizing systemic side effects, and improving therapeutic outcomes in diverse biomedical applications [23].

Physiological stimuli-responsive drug delivery systems, also known as “smart” or “intelligent” systems, are designed to release their therapeutic payload in a controlled manner in response to specific biological cues within the body. This approach helps deliver drugs precisely to a target site, such as a tumor or an inflamed area, thereby maximizing efficacy and minimizing side effects. Physiological stimuli are divided into:

1. pH-Responsive systems, which exploit the natural pH gradients in the body, e.g., pH 2–3 in the stomach, pH 6.5–7.4 in the small intestine [50]. For this purpose, pH-sensitive polymers or nanocarriers are often used. These materials contain ionizable groups (e.g., carboxyl or amino groups) that undergo protonation or deprotonation in response to a pH change. This change alters the polymer’s solubility or structure, causing it to swell, dissolve, or undergo a conformational change that results in releasing the encapsulated drug.
2. Temperature-responsive systems are triggered by changes in temperature during, e.g., fever, inflammation, or tumors, or are induced externally (e.g., hyperthermia therapy). The most common materials are thermo-responsive polymers that undergo a reversible phase transition at a specific temperature, known as the lower critical solution temperature. Below this temperature, the polymer is hydrophilic and swells with water, but above it, it becomes hydrophobic and shrinks, expelling the encapsulated drug [51].
3. Enzyme-responsive systems are based on using specific enzymes that are overexpressed or uniquely present in certain diseases. The drug is often linked to the carrier via a bond that can be specifically cleaved by a target enzyme. When the system encounters the enzyme at the diseased site, the enzyme degrades the carrier or breaks the linker, thus releasing the drug [52].
4. Redox-responsive systems are designed to respond to the differences in redox potential between healthy and diseased cells. The drug carrier is cross-linked with a redox-sensitive bond, most commonly a disulfide bond (-S-S-). This bond remains stable in the oxidative extracellular environment but is cleaved by the high concentration of glutathione inside the target cell, e.g., in a tumor. The cleavage leads to the degradation of the carrier and the rapid release of the drug [53].

**Figure 4:**
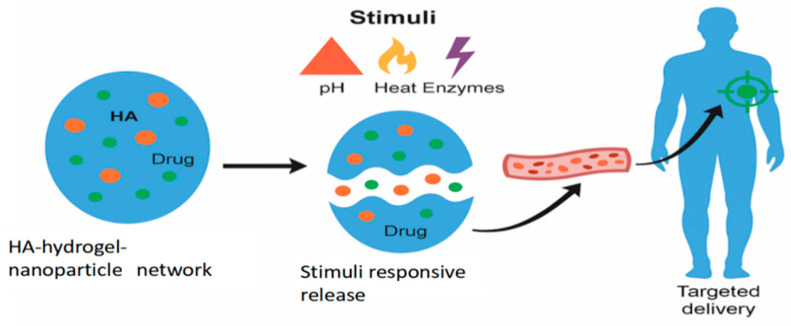
HA-based hydrogel–nanoparticle composites for smart drug delivery. Drugs encapsulated within the HA hydrogel–nanoparticle network are released in response to specific physiological stimuli such as changes in pH, temperature, or enzymatic activity.

As shown in Figure 4, NPs loaded with therapeutic agents are embedded within an HA hydrogel matrix, creating a multifunctional platform that combines the high loading capacity of NPs with the biocompatibility and tunable properties of hydrogels.

## 4. Treatment of Skin Wounds

The availability of medications to promote wound repair is still restricted, despite the significant need for wound repair agents in clinical use. Since the market’s demand exceeds what the industry can supply, researchers, the general public, and the healthcare sector are highly interested in the discovery and application of efficient wound repair agents, particularly those of natural origin [54]. Many commercially available products, including those based on natural biopolymers, stem cells, and microRNAs, are currently being used to heal wounds [55].

Wounds are characterized as disruptions to the continuity of the skin, tissues, and mucous membranes caused by physical or thermal damage. They are classified as either acute or chronic wounds. Obviously, depending on the size and depth of the wounds, acute wounds, like skin or surgical wounds, heal completely in a few weeks [56,57,58,59]. In contrast, chronic wounds, including pressure, leg, and diabetic ulcers and severe burns, are more difficult to manage due to their slow healing time, abnormal healing process, and persistence. Since the number of people with chronic wounds is predicted to rise as the population ages, it is crucial to address chronic wounds as a long-term problem. Dry wound dressings, such as gauze and bandages, are commonly employed during the initial phases of wound healing. Nonetheless, these dressings are difficult to provide a moist environment, which is critical for retaining wound exudates and promoting effective wound healing. Additionally, they have a propensity to adhere to wounds, which can result in complications like inflammation, wound rupture, and discomfort when removed. Consequently, the newly formed skin or tissue can be compromised, potentially causing re-injury and bleeding. Modern dressings can overcome the limitations of dry wound dressings by providing an ideal temperature and humidity to facilitate wound healing. Moreover, they also have non-adhesive properties to minimize the pain during dressing changes for patients. They include various types, such as films, hydrocolloids, hydrogels, foams, and alginates [59,60].

Approximately 50% of the total HA resides in the skin, both in the dermis and the epidermis [37]. Hyaluronan is responsible for maintaining the structural integrity of the ECM and tissue hydration and plays an important role in the wound-healing process [46,59]. The general process of wound healing involves a series of sequential phases, including hemostasis, inflammation, proliferation, remodeling, and reepithelization [61]. During the hemostasis phase, high-molecular-weight (HMW) HA accumulates within the wound bed and then binds to fibrin and fibronectin, forming a temporary scaffold that facilitates the accumulation of fibroblasts and inflammatory cells such as lymphocytes, neutrophils, and macrophages.

The synthesis of HA significantly increases during the inflammatory phase. The unique properties of HA, particularly its hydrophilicity, allow for the passive diffusion of water into the interstitial space. As a result, edema forms around the wound, and chemotaxis of inflammatory cells occurs. Low-molecular-weight (LMW)-HA signals through receptors, especially Toll-like receptor 4 (TLR4), which is present on the surface of immune cells such as macrophages and dendritic cells. The binding of LMW-HA to TLR4 triggers a signaling cascade inside cells, which leads to the activation of transcription factor nuclear factor kappa B (NF-κB). NF-κB then translocates to the nucleus and promotes the transcription of genes that encode for proinflammatory cytokines, including tumor necrosis factor-α (TNF-α), interleukin-1β (IL-1β), and IL-6. These cytokines then amplify the inflammatory response, recruiting more immune cells to the site of injury [62]. Inflammation affects the dilation of blood vessels, leading to redness of the skin and increased warmth at the site of the injury. Inflammation and its associated symptoms are essential for successful wound repair [46]. Conversely, HA also plays a role in reducing and moderating the inflammatory response through its interaction with the hyaladherin TNF-stimulated gene-6 [43,63].

In the proliferative phase, fibroblasts migrate from the dermis to the site of the wound, which is promoted by small fragments of HA and growth factors, which serve as signaling molecules [46,63]. Small HA fragments (oligosaccharides) and wound growth factors cooperate to attract and mobilize fibroblasts by: 1) directly engaging HA receptors (CD44, RHAMM) and innate receptors (TLR2/4) to trigger cytoskeletal, focal-adhesion and mitogen-activated protein kinase/phosphoinositide 3-kinase (MAPK/PI3K) signaling that drives chemotaxis, 2) inducing local production and activation of wound cytokines (for example TGF-β) that change fibroblast phenotype and directional motility and 3) altering ECM structure and growth-factor availability (increasing matrix porosity and concentrating/releasing fibroblast growth factors and platelet-derived growth factor, which together lower the physical and biochemical barriers to migration [64]. Subsequently, fibroblasts produce glycosaminoglycans and collagen that facilitate ECM remodeling. Hyaluronan has a significant impact on neovascularization as well. The involvement of HA in angiogenesis depends on its molecular weight [46,63]. The HMW HAs inhibit angiogenesis, whereas LMW HAs (6–20 kDa) exhibit proangiogenic effects [65]. Additionally, LMW HA supports the phase of forming epidermis. This function is related to the interaction of CD44 in endothelial cells with HA oligomers, which is critical for stimulating the differentiation of keratinocytes as the main cells of the epidermis. CD44 is highly expressed on keratinocyte membranes, and its binding to HA activates intracellular signaling pathways, such as the RhoGTPase and calcium mobilization pathways. This signaling promotes the transition of keratinocytes from a proliferative basal state to a differentiated state, which is necessary for forming the multiple layers of the epidermis [66]. Activated endothelial cells stimulate the processes of migration, granulation, and the formation of new blood vessels [46,63].

**Figure 5:**
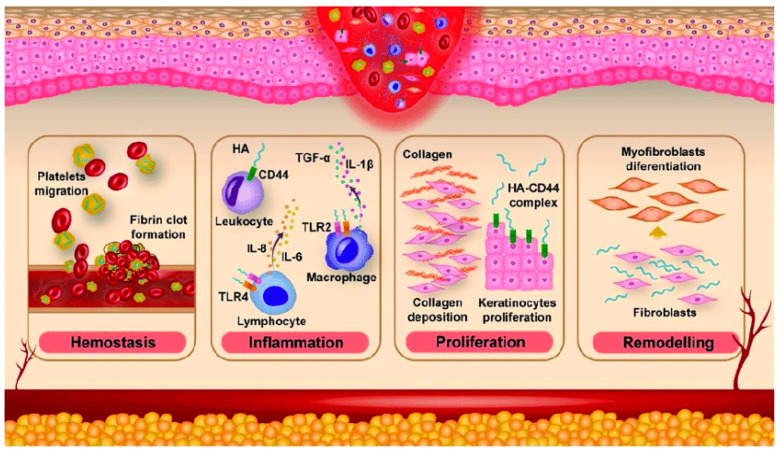
The involvement of hyaluronan in the phases of skin wound healing. Reprinted with permission from Graca et al. [69]. Copyright 2025 Elsevier.

In the remodeling phase, HA primarily integrates with CD44. The activation of CD44 stimulates cells derived from mesenchyme, referred to as myofibroblasts, which can produce ECM components. Primarily, myofibroblasts are specialized cells that are responsible for the contraction of a wound, which helps facilitate the closure of a wound [46,63]. They possess a contractile apparatus similar to smooth muscle cells, containing bundles of actin and myosin [67]. Moreover, they are responsible for the production of HA, most often with a molecular weight of approx. 480 kDa. LMW HA enhances the expression of transforming growth factor (TGF)-β1 and TGF-β2, which are responsible for scar formation. On the other hand, HMW HA increases the expression of TGF-β3, which is involved in inhibiting the scarring process [46,63]. LMW-HA is pro-inflammatory and pro-angiogenic. It binds to CD44 and triggers the recruitment of immune cells and stimulates the formation of a stiff, fibrous scar. LMW-HA contributes to the fibrotic, scarring process by signaling for an exuberant and disorganized deposition of collagen. In contrast, HMW-HA has anti-inflammatory and anti-scarring properties. It acts as a structural scaffold, maintaining a hydrated, open space in the ECM that supports cell migration and tissue regeneration. By binding to CD44, it can inhibit the inflammatory signaling pathways. Its presence creates an environment conducive to organized tissue repair, similar to the process seen in fetal skin, which heals without scarring [68]. Figure 5 illustrates the phases of skin wound healing.

The wound site is an optimal environment for microbial growth, and the proliferation of microorganisms at the injured site delays the healing process. Reducing microbial infection is, therefore, crucial for effective wound recovery. For this reason Yao et al. [70] developed Ag NP@chitosan@β-1,3-glucan/HA with high biocompatibility, hemocompatibility, promotion of blood coagulation and wound healing. The results of the in vitro cell migration test showed that after 8 h of incubation, approx. 59% of the damaged area healed due to the migration and proliferation of NIH/3T3 cells into the scratched region (control group). In contrast, cells treated with Ag NP@chitosan displayed approx. 54.0% of the damaged area healed, indicating the toxicity of Ag NP@chitosan (Ag concentration of 25 ppm). The wound contraction in the Ag NP@chitosan@β-1,3-glucan/HA-treated group reached 68.6% compared with the control group. The results of antibacterial activity showed that the inhibition of *E. coli* growth followed the order AgNO_3_ (98%) > Ag NP@chitosan (73%) > AgNP@chitosan@β-1,3-glucan/HA (68.6%). Similar results were observed for *S. aureus* growth inhibition. The authors suggested that the presence of chitosan and β-1,3-glucan/HA restricted ionic silver release and diffusion, thereby reducing the antibacterial effect on *E. coli* and *S. aureus*. Hong et al. [71] fabricated “sea urchin”-shaped Cu@SiO_2_ NPs that were incorporated into catechol-modified and photopolymerizable HA hydrogels. The results indicated that the control hydrogel (without SiO_2_) demonstrated antibacterial effectiveness as follows: *K. pneumoniae* (63.8%), MRSA (methicillin-resistant *S. aureus*, 91.1%), and *S. mutans* (99.9%). Both Cu@SiO_2_ and Cu@SiO_2_-hydrogels exhibited a significant bactericidal rate of 99.9% against all three bacterial strains. Cu@SiO_2_-hydrogel had antibacterial activity even at a concentration of 2 g/mL. Gonçalves et al. [72] fabricated bacitracin-loaded photo-crosslinked methacrylated hyaluronan (HAMA)/chitosan NPs. These NPs showed significant antibacterial activity against *S. aureus*, MRSA, and *S. epidermis* and demonstrated inhibition of biofilm formation and a positive effect on the proliferation of L929 cells. In another study, Khachatryan et al. [73] fabricated an innovative HA/ozonated olive oil rheologically stable hydrogel, which was demonstrated to have an antimicrobial effect against skin bacteria and pathogenic Candidas and no cytotoxicity in the HaCat human keratinocyte cell line.

Wang et al. [74] explored the application of novel hydrogels in the treatment of psoriasis. The authors prepared HA-cholesterol nanohydrogels (NHs) and NH-carbopol formulations for dermal delivery of betamethasone. Both in vitro (using Strat-M^®^ membrane) and ex vivo (using pig ear skin) studies indicated that these hydrogels significantly enhanced skin permeation and drug retention in the deeper layers of both epidermis and dermis. Moreover, the NHs showed potential antipsoriatic activity by downregulating the proinflammatory cytokines in human keratinocytes (HaCaT cell line) and in an ex vivo 3D human skin tissue model (EpiDerm-FT™). In another study, Zeng et al. [75] improved the treatment of psoriasis by focusing on the preparation of polyvinyl alcohol (PVA) microneedles that incorporated self-assembled HA/methotrexate NPs, resulting in reduced adverse effects. Upon transdermal delivery using polymeric microneedles, the HA-based therapeutic NPs targeted the inflammatory skin cells by interaction of the HA group with the CD44 protein that is highly expressed on the cell membrane in the psoriatic skin. In the imiquimod-induced psoriatic mice, only one application of the polymeric microneedle dressing resulted in lower epidermal hyperplasia and reduced expression of inflammatory factors. The release of methotrexate from HA-methotrexate NPs lasted for over 7 days, compared to the rapid dispersion of free methotrexate at pH 5.5 or a slight release of methotrexate at pH 7.4. Zhang et al. [76] demonstrated a significant improvement in methotrexate’s transdermal penetration and retention within psoriatic lesions due to the HA-modified liposomes. Further, methotrexate loaded into HA/silk NPs also effectively attenuated psoriasis-induced inflammatory responses. After 48 h, the accumulated amounts of methotrexate released from liposomes were 43.7 ± 1.62% and from liposomes/HA and 34.65 ± 1.25%. The latter value is linked with the fact that HA reduced membrane permeability and bilayer fluidity. In another study, Cheng et al. [77] fabricated methotrexate/HA/silk NPs, which effectively attenuated psoriasis-induced inflammatory responses. The inclusion of HA improved the targeting of NPs, especially by interacting with overexpressed CD44 proteins in psoriatic skin, which resulted in a greater accumulation of methotrexate at inflammation sites and thus enhanced the drug’s therapeutic efficacy. Xu et al. [78] fabricated biocompatible and anti-inflammatory hydrogel composed of oxidized HA and carboxymethyl chitosan with encapsulated *Resina draconis* particles. The results of in vitro experiments confirmed that the gel was biocompatible and enhanced L929 cell migration and inhibited the production of inflammatory cytokines. It also induced rapid hemostasis in rats, downregulated the levels of inflammatory cytokines, and promoted collagen regeneration, thereby accelerating the rebuilding of skin structures and wound recovery.

Developing sustainable and effective 3D skin models for investigating inflammatory skin biology remains a challenge. For this reason Xu et al. [79] incorporated halloysite nanotubes into a composite collagen/alginate/HA hydrogel to form a novel 3D skin model, which enhanced the differentiation and adhesion of keratinocytes and fibroblasts and promoted wound healing in C57BL/6 mice. Moreover, these hydrogels had similar viscoelastic and mechanical properties to natural skin.

In the realm of skin wound treatment, numerous innovative wound dressings have been developed. Exosomes (Exos), which are naturally derived NPs containing bioactive molecules, represent ideal cell-free therapeutic modalities. However, challenges related to delivery, long-term preservation, and activity maintenance of exosomes limit their application [80]. Yang et al. [80] proposed the use of hydrogels for the encapsulation of human umbilical vein endothelial cell mesenchymal stem cell (HUVECs MSC)-derived exosomes combined with an antimicrobial peptide DP7 into HA hydrogel, which facilitated tissue regeneration and inhibited scar formation in mice with deep second-degree burn infection. Healing was mediated through miR-21-5p (onco miRNA). To treat skin inflammatory diseases, Politi et al. [81] demonstrated that the piperine-loaded NPs incorporated into HA/alginate-based biomembranes showed attenuated inflammation in mouse ear edema up to 43.6% and no cytotoxicity. Zhu et al. [82] prepared a controlled-release 3D adipose-derived MSc-derived exosomes HA hydrogel for burn wound healing. When compared with 2D-Exos and 2.5D-Exos, 3D-Exos promoted HACATs and HUVECs proliferation and migration more significantly. Additionally, 3D-Exos had stronger angiogenesis-promoting effects in tube formation of HUVEC cells. Dong et al. [83] delivered adipose-derived stem cells for the treatment of burns. The authors used poly(ethylene glycol) diacrylate (hyperbranched-PEGDA) hydrogel, thiol-based HA, and an arginylglycylaspartic acid (RGD) peptide, which significantly accelerated wound closure and neovascularization, reduced scar formation, and protected the implanted cells from the detrimental wound environment associated with burns.

## 5. Treatment of Diabetes Mellitus

Diabetes mellitus affects approx. 8% of the population. It is characterized by abnormal glucose metabolism, which can lead to complications such as renal failure, retinopathy, atherosclerosis, peripheral vascular disease, neuropathy, and impaired wound healing. Hyperglycemia, i.e., high blood glucose levels, results from the loss of normal insulin response from target cells such as muscle and fat cells in type 2 diabetes and the loss of pancreatic insulin-producing b cells in type 1 diabetes, which is an autoimmune disease [84,85], whereas autoreactive T lymphocyte cells play an important role in this process. As a consequence, patients with type 1 diabetes can be treated only with exogenous insulin supplementation, which may mitigate or delay, but not eliminate, the risk of developing secondary complications associated with the disease.

The solution is the transplantation of pancreatic islets for type 1 diabetes. However, their transplantation from cadaveric donor organs has been associated with limited clinical success in few centers, mainly due to either the need for lifelong immunosuppression of the recipients, with its imminent, related complications, or the restricted availability of cadaveric human donor pancreases [86,87]. Recently, the aim has been to use isolated cells with an immune-protective shield to prevent physical contact between the transplanted cells and the host’s immune system while maintaining the microcapsules’ biocompatibility and physical–chemical properties over time. Especially, this revolutionary approach was applied to islet grafts in diabetic recipients who are not immunosuppressed at a preclinical (in rodents) and subsequently clinical level. Alginic acid-based polymers proved to be superior to all other polymers and potentially suitable for microencapsulation of live cells due to their immunoselectivity, biocompatibility, and favorable permeability in the following decades. In fact, only alginic acid-based microcapsules containing allogeneic islets ultimately entered pilot human clinical trials in patients with type 1 diabetes mellitus [86,87,88,89,90]. Chemically modified alginate formulations can allow for the long-term transplantation of islets to eliminate insulin deficiency [91]. Embryonic and adult stem cells loaded into microcapsules provide an unlimited source that could prevent the shortage of donors to complete differentiation into insulin-producing cells [92].

Besides alginic acid, there is also an interest in using HA in the treatment of diabetes mellitus. To prevent rejection of the transplanted islets, Scheiner et al. [93] introduced a novel way to encapsulate them in biomaterial implants, which can protect the islets and provide an organ-like environment. The authors pursued a prevascularization strategy by incorporation of vascular endothelial growth factor (VEGF)-loaded microspheres in 3D printed poly(dimethylsiloxane)-based devices prior to their prospective loading with transplanted cells. Microspheres (~50 m) were based on poly(ε-caprolactone-PEG-ε-caprolactone)-b-poly(l-lactide) multiblock copolymers and were loaded with 10 mg VEGF/mg microspheres and subsequently dispersed in an HA carrier liquid. In vitro release studies demonstrated continuous release of fully bioactive VEGF for 4 weeks. Wang et al. [94] produced 3D-printed islets by integrating pancreatic ECM and HAMA as specific bioinks. This hydrogel enhanced insulin levels and islet function and activity in C57BL/6J mice. Further, it maintained normal blood glucose levels for 90 days and rapidly secreted insulin in response to blood glucose stimulation, which can facilitate growth of new blood vessels. The islet organoids constructed by 3D printing can mimic the microenvironment of the pancreas and maintain islet cell adhesion and morphology through the Rac1/ROCK/MLCK signaling pathway, thereby improving islet function and activity. Additionally, the 3D-printed structures were appropriate for the formation of new blood vessel networks, bringing hope for the long-term efficacy of islet transplantation. In another study, Li et al. [95] fabricated a hydrogel containing methacrylated gelatin (GelMA) and methacrylated heparin, which can deliver VEGF in a sustained manner, thereby inducing subcutaneous angiogenesis. In addition, islet-laden microgels using HAMA/PEGDA/carboxybetaine methacrylate hydrogels provided a favorable microenvironment for islets and simultaneously inhibited host immune rejection via anti-adhesion of proteins and immunocytes. The bioartificial pancreas demonstrated the capacity to reverse blood glucose levels in diabetic mice from hyperglycemia to normoglycemia for at least 90 days.

Chronic nonhealing skin wounds are significant complications associated with diabetes, with a high morbidity, and they can lead to disability or death. Conventional drug therapy is ineffective due to drug-resistant bacterial infections, oxidative stress, and immune dysfunction. Multifunctional hydrogels with antibacterial, antioxidant, and anti-inflammatory properties are becoming an emerging trend in their treatment. However, to meet all demands has been a challenge so far [96,97]. It was demonstrated that high levels of hemoglobin A1c, which is an indicator of poor hyperglycemic control, correlated directly with delayed wound healing. Moreover, neuropathy also results in the formation of non-healing skin ulcers in the feet and lower limbs, which is often a problem in diabetic patients [68].

Impaired wound healing in diabetic patients is mechanically linked to hyperglycemia through the HA synthesis and degradation of HA in the pericellular lining of endothelial cells (glycocalyx). In this process leukocyte recruitment is increased, a proinflammatory microenvironment is formed, and thrombosis in vessels is exacerbated by platelet-derived hyaluronidase that adversely affects not only blood vessel function but also adjacent pericytes, fibroblasts, and smooth muscle cells [85,98]. Further, the loss of the endothelial glycocalyx in diabetes leads to reduced nitric oxide production. Hyperglycemia impairs the ability of the endothelial glycocalyx to regulate the permeability of macromolecules [98].

To enhance the treatment of diabetic wounds, numerous novel wound dressings were designed. Recently, Alissa et al. [99] designed a silk/HA (SH) scaffold and the SH scaffold with incorporated curcumin (SCN) NPs. The results of experiments in diabetic rats showed that regeneration, including wound closure, fibroblast and blood vessel counts, collagen density, tensile strength, and concentration levels of TGF-β and VEGF in SH and SCN groups, was significantly higher compared to the control group. These changes were more obvious in the SCN ones (*p* < 0.05). In contrast, the number of neutrophils and macrophages and the levels of TNF-α and IL-1β decreased more significantly in the SCN group than in the control and SH groups. Ma et al. [100] developed a multifunctional HA-based material capable of serving as either an injectable wet microgel or dry microspheres. Initially, they engineered Fe^2+^/tea polyphenol metal–polyphenol network-functionalized HAMA microspheres. These particles were found to suppress inflammation and facilitate scavenging of reactive oxygen (ROS). A deferoxamine (DFO)-loaded zinc-based metal–organic framework (ZIF-8@DFO) was then coated using phenylboronic acid (PBA)-functionalized α-polylysine to produce NPs with antibacterial and pro-angiogenic properties. Liu et al. [101] prepared quaternary ammonium chitosan/dihydrocaffeic acid/l-arginine and oxidized HA-dopamine self-healing hydrogel. Methacrylated PVA and PBA microneedles and gallium porphyrin modified with 3-amino-1,2-propanediol and insulin were encapsulated at their tips. These self-healing, dual-layer, drug-carrying wound dressings exhibited excellent biocompatibility, slow drug release, and antimicrobial properties. Pu et al. [102] prepared a material that integrated an anhydride and methacrylic-modified HA hydrogel (AHAMA) and chitosan NPs encapsulating zinc-based polymetallic oxonate nanozyme (Zn-POM) and glucose oxidase (GOx), facilitating a sustained release of both enzymes. The GOx alleviated hyperglycemia during wound healing. Zn-POM mimicked catalase and superoxide dismutase activities, was anti-inflammatory, and enhanced angiogenesis and collagen regeneration within wounds. In a rat diabetic wound model, the application of AHAMA/CS-GOx@Zn-POM enhanced neovascularization and collagen deposition, accelerating wound healing. In another study, Li et al. [103] fabricated an innovative hydrogel with bactericidal and anti-inflammatory properties by crafting a pH/ROS-responsive scaffold from PBA-grafted HA and polyethyleneglycol (PEG)-dopamine (4A-PEG-dopamine) infused with the antimicrobial peptide (AMP) and ROS-sensitive micelle mPEG-TK-poly(lactic-co-glycolic acid) (PLGA) loaded with quercetin, which, after the release from micelles, alleviated oxidative stress and enhanced the polarization of macrophages M2 via the Akt/STAT6 signaling pathway. Liu et al. [104] prepared the Ag@Pt-Au-lysozyme/HA-LL-37 formulation, which was effective in targeting enzyme-catalyzed, photothermal, and chemodynamic therapy to kill bacteria (*E. coli*, *S. aureus*, and *B. subtilis*) and promoted the healing of diabetic wounds. In other studies Luo et al. [105] proposed an ε-polylysine/MnO_2_ nanozymes/gellan gum/HA-based multifunctional hydrogel. Wu et al. [106] prepared microneedles composed of carvacrol, cyclodextrin, mesoporous ceria NPs, and HA. Yang et al. [107] formed glycyl methacrylate gelatin, oxidized HA, and lauric acid hydrogel. Gao et al. [108] formed the HAMA-MnO_2_–Au–mSiO_2_@aFGF Janus NP hydrogel. All these hydrogels significantly facilitated skin wound healing in vivo. Wu et al. [109] developed multifunctional HAMA/carboxymethyl basic fibroblast growth factor-encapsulated chitosan microneedles with alginate to maintain the moisture balance in the wound exudate. They accelerated angiogenesis, immunity modulation, and collagen synthesis.

To maintain stable normal glucose levels for 6 h in diabetic mice, Hua et al. [110] prepared silk fibroin/HA hydrogel microneedles. The extensive proliferation and well-distributed network of human HUVECs on the microneedles’ surface underscored the high cytocompatibility and cell viability of the microneedles. In another study Huan et al. [87] fabricated the alginate and HAMA microfibers loaded with pancreatic α- and β-cells, which were able to maintain the capacity of dual-mode glucose responsiveness attributed to the glucagon and insulin secreted by the encapsulated pancreatic α- and β-cells. After transplantation into the brown adipose tissue, these cell-loaded microfibers provided successful blood glucose control in rodents and prevented hypoglycemia. Lu et al. [111] fabricated two types of reverse micelles: a self-emulsifying drug delivery system and an oil-in-water HA formulation, both of which could reduce blood glucose in type 2 diabetic rats, protect pancreatic β cells to some extent, and alleviate insulin resistance and hyperlipidemia complications while ensuring good safety. In another study, Tu et al. [112] modified HA chains with vinyl sulfone and thiol functional groups, which allowed for precise glucose and ROS regulation by encapsulating glucose oxidase, horseradish peroxidase, and tannic acid within the HA hydrogel.

To treat diabetic retinopathy (DR), one of the novel potent anti-vascular endothelial growth factors is apatinib (Apa). Topical administration of Apa-loaded bovine serum albumin (BSA)/HA NPs showed neither cytotoxicity on rabbit corneal epithelial cells nor on the DR rat model. The treatment resulted in alleviated retinal micro- and ultrastructural changes in the eyes treated with topical HA-Apa-BSA-NPs, showing a normal basement membrane and retinal thickness comparable to that of the normal control and intravitreally injected NPs [113]. 

To improve diabetic nephropathy, the HA-functionalized span-labrasol nanovesicular transdermal therapeutic system of ferulic acid significantly decreased the levels of blood glucose, creatinine, and intercellular adhesion molecule-1 levels. In contrast, the levels of insulin, AMP-activated protein kinase, and sirtuins were increased [114].

## 6. Treatment of Eye Diseases

To treat corneal and conjunctival disorders, over 90% of ophthalmic drugs are used as eye drops or ointments. However, physiological barriers such as nasolacrimal drainage, lacrimation, blinking, and anatomical barriers limit the drugs’ intraocular penetration and bioavailability. About 30 µL of fluid can be retained in the eye’s inferior cul-de-sac, with excess drops being released following the first blink. Every five minutes, the tear film is renewed, making it easier to remove the unabsorbed drug. The cornea’s surface consists of five to six epithelial layers, and their tight connections prevent paracellular drug transfer. It is challenging for hydrophobic medications to penetrate through the hydrophilic corneal stroma and into the anterior chamber. Like the corneal epithelium, the conjunctiva acts as a barrier to hydrophilic medications, even though scleral penetration is more practical. Because of these obstacles, less than 5% of topical ocular medicine is absorbed. Age-related macular degeneration (AMD) is one of the ocular conditions that topical medication application frequently fails to treat.

High-molecular-weight monoclonal antibodies that hardly penetrate the cornea and sclera topically, as well as anti-VEGF drugs, must be administered intraocularly regularly to patients with exudative AMD. Frequent administration of these injections raises the risk of infection and expenses. Intraocular implants, which release corticosteroids for months to years with only a single injection, have been designed to avoid these issues [115,116,117,118]. Additionally, long-term anti-VEGF drug implants have been designed [119]. The goal of developing drug delivery systems in ophthalmology is to effectively administer drugs in the least invasive way possible. Another goal is to use slow-release strategies to extend the dosage interval if an invasive procedure is required. This method improves therapy adherence, lessens socioeconomic expenses, and reduces patient suffering [120,121].

Non-invasive topical formulations that can be self-administered in non-clinical settings include eye drop formulations, ophthalmic gels, and therapeutically soaked contact lenses. On the other hand, corneal retention time and therapeutic bioavailability at the target site are significantly decreased by selective inhibition of deeper intraocular therapeutic permeation through the static anatomical barriers in conjunction with precorneal clearance mechanisms like the upregulation of tear film clearance via nasolacrimal drainage and reflex blinking. While therapeutic bioavailability within the retinal tissues is heavily reliant on the physicochemical characteristics (molecular radius, lipophilicity) of the drug itself, only 3–5% of topically applied medications typically penetrate the cornea and are available for further migration into the posterior intraocular tissues. It may be necessary to administer the drug more often and repeatedly in order to gain a therapeutic response. These factors could lead to the drug being administered irregularly and in a way that differs from the approved administration regimen.

To address the drawbacks of conventional ophthalmic formulations, polymeric ocular drug delivery systems (ODDs) have been developed, including hydrogels, nanoformulations, and biological stimuli-responsive systems. ODDs have been developed using both synthetic and biological polymers. Moreover, the former have benefits of biomimetic cell receptor targeting and improved biocompatibility due to rapid in vivo enzymatic degradation, while the latter have demonstrated increased mechanical strength and stability in vivo [121,122,123].

Hyaluronan is present in all ocular tissues, i.e., the iris, lenses, vitreous body, retina [35,104], lacrimal glands, corneal epithelium, and conjunctiva. It has also been found in human tears through the cell surface glycoprotein CD44 [124]. Hyaluronan possesses mucoadhesive properties. It adheres to the corneal mucin layer through non-covalent bonds, where its acid groups interact with sialic acid in eye mucin [43,121]. Furthermore, HA was shown to stimulate corneal epithelial cell migration, which plays a role in corneal wound healing. It is supposed that HA also enhances water retention on the corneal surface. Hyaluronan also provides the required viscosity of the vitreous body [44]. Due to its hydrophilic and viscoelastic properties, HA slows down the evaporation of tears, reduces irritation and friction, and hydrates eyes. For this reason, HA is used in formulations for artificial tears that are used to treat dry eyes [45,125,126].

Additionally, HA can be combined with other substances like triglycerides, phospholipids, vitamin B12, coenzyme Q10, hydroxypropyl guar, antibiotics, or steroids to increase the thickness of the tear film, sustain the ocular surface, reduce DES symptoms, and mitigate oxidative stress in the conjunctival epithelium of DES patients [124]. Furthermore, HA is extensively used in drug delivery systems, contact lenses, vitreous substitutes, corneal wound healing [43,45,121,125,126], and ocular surgery, where it creates and preserves ideal circumstances to encourage the healing of the surgical site. This process is accomplished by adjusting intraocular pressure, reducing the chance of adhesions, and stabilizing the tear film. Additionally, it is frequently utilized in procedures involving the anterior portion of the eyes, including corneal plastic surgery, trabeculectomy, glaucoma therapy, refractive surgery, and cataract removal [125,127].

To reduce the drug degradation, HA-based NPs penetrate ocular cells and interact with the corneal epithelium. Prolonged ocular residence time, improved lubrication, drug absorption and stability, and intraocular penetration are benefits of using HA-based NPs [43,121,124]. Additionally, HA binds to the cellular receptor CD44, rendering HA-NPs a suitable vector for gene therapy. Intravitreal injections can be used for the administration of drugs, gene therapy, or the artificial vitreous body. These injections can penetrate into barriers that affect drug absorption, such as corneal tissue, tear flushing and secretions, or drug delivery to the target area located in the posterior eye [121].

To reduce the dryness and the dosing frequency, Sakpal et al. [128] prepared chitosan, PVA, and HA nanofibers encapsulating dexamethasone sodium phosphate (DSP), which helped mucoadhesion and lubricated the cornea. The drug-loaded electrospun nanofibers were set between two blank nanofiber scaffolds. The outer nanofiber scaffolds composed of HA (0.8% *w*/*v*) and PVA (10% *w*/*v*) provided mucoadhesion and lubricated the cornea to reduce eye dryness. The middle layer of the insert was fabricated using chitosan (1% *w*/*v*), PVA (10% *w*/*v*), and DSP (0.1% *w*/*w*). Further, Ngo et al. [129] prepared NPs containing HA/cystamine/oleic acid/cyclosporine A (a drug for the treatment of multifactorial DES). These NPs increased in vitro drug permeation compared to Restasis^®^, effectively inhibited VEGF-induced endothelial cell proliferation, augmented macrophage polarization into the M2 phenotype, and downregulated the expression of proinflammatory cytokine levels in lipopolysaccharide-induced M1 macrophages. Grimaudo et al. [130] designed an innovative ophthalmic remedy composed of HA nanofibers for the dual delivery of ferulic acid and antimicrobial peptide ε-polylysine, whereas both the blank and nanofibers had antibacterial effects against *P. aeruginosa* and *S. aureus*. The antibacterial mechanism of ε-polylysine against the selected bacteria has been attributed to membrane disruption related to the interactions of the primary amine surface groups with the cell membrane.

Wounds and chemical burns to the cornea can often result in a loss of corneal epithelium function and cause severe damage to the corneal stroma. Injectable hydrogels have been widely applied in corneal tissue engineering because they are easy to handle and can completely fill the defect area with minimally invasive surgical procedures [131]. In the treatment of corneal injuries, HA is commonly used, which helps maintain the moisture of eye tissues and reduces scarring and inflammation [132]. Ribeiro et al. [133] developed alginate/HA lipoplexes NPs, which protected and delivered siRNA molecules targeting caspase-3 into the retina. Liu et al. [132] improved the solubility and bioavailability of proanthocyanidins by preparing HA/proanthocyanidin NPs. They had antioxidant and anti-inflammatory properties in vitro. Studies on C57 mice confirmed faster healing of corneal injuries and reduced corneal opacity. Wang et al. [131] developed a double-network biocompatible hydrogel based on GelMA and oxidized HA for the reparation of focal corneal defect in rabbits to lower the risk and complications of penetrating keratoplasty and lamellar keratoplasty and to meet the demand for donated corneas. Huang et al. [134] fabricated HA-based liposomes loaded with retinal cell-targeted ginsenoside Rg3, which significantly attenuated the oxidative stress induced by retinal ischemia–reperfusion injury and promoted the transition of M1-type macrophages to the M2 type. Liposomes can regulate SIRT/FOXO3a, NF-κB, and STAT3 signaling pathways. Jin et al. [135] prepared a nanogel composed of polyethylenimine-benzene boric acid-HA loaded with cerium oxide nanozyme with CX3CL1 protein for targeting the posterior segment of the eyes. The nanogel targeted immunomodulation and retinal protection in uveitis models.

Retinal neovascularization (RNV) is a typical feature of ischemic retinal diseases that can lead to traction retinal detachment and even blindness in patients, in which VEGF plays a pivotal role. However, most anti-VEGF drugs currently used for treating RNV, such as ranibizumab, need frequent and repeated intravitreal injections due to their short intravitreal half-life, which increases the incidence of complications [136]. Duan et al. [136] prepared an aminated HA/aldehyde-functionalized pluronic 127/ranibizumab hydrogel. In the rabbit persistent RNV model, the hydrogel continuously released ranibizumab for more than 7 weeks and had excellent anti-angiogenic efficacy by decreasing vascular leakage and neovascularization within 12 weeks. Miyagawa et al. [137] designed gelatin-epigallocatechin-3-gallate self-assembled NPs with HA conjugated with arginine-glycine-aspartic acid to be used for targeted therapy in corneal NV. These NPs significantly reduced the formation of endothelial cell tubes of pathological vessels in the mouse cornea after chemical cauterization, reduced both VEGF and MMP-9 protein in the NP-treated cauterized corneas, and inhibited metalloproteinase (MMP)-2 and MMP-9 activity in HUVECs. These NPs, as eye drops, can reduce the dosing frequency due to the advantages of NPs’ interaction with the ocular surface to achieve higher drug bioavailability.

For the treatment of neovascular AMD Durak et al. [138] investigated the anti-VEGF peptide HRH, which has high affinity to the VEGF-Fc receptor and was used as the bioactive agent to control neovascularization of the retina. The HA nanogel was formed by incorporating divinyl sulfone and cholesterol to increase the stability and control the size of the nanodrug. The nanogel was not toxic to ARPE-19 cells, whereas it inhibited HUVEC proliferation due to the anti-VEGF peptide in the nanogel structure. In vivo experiments with a chick chorioallantoic membrane showed that nanogel had higher antiangiogenesis activity compared to free HRH. Additionally, in an oxygen-induced retinopathy model in mice, the excessive growth of blood vessels was notable.

Vitreous substitutes are used in clinics to maintain retinal apposition and preserve retinal function. Since most of them are oils and gases, their use has disadvantages, including the need for subsequent surgical removal and strict face-down positioning post-surgery. The authors designed a novel vitreous substitute made of HA-oxime crosslinked hydrogel. The hydrogel was cytocompatible in vitro with photoreceptors from mouse retinal explants and biocompatible in rabbit eyes. The ocular pressure in the rabbit eyes was consistent over 56 days, demonstrating minimal or no swelling. The implanted hydrogel did not impact retina function using electroretinography over 90 days versus eyes injected with balanced saline solution [139].

In glaucoma treatment, Brugnera et al. [140] fabricated latanoprost-loaded phosphatidylcholine liposomes with HA (LAT-HA-LIP) to extend the hypotensive effect of latanoprost while protecting the ocular surface. The results showed that the potential of LAT-HA-LIP had a hypotensive effect. LAT-HA-LIP exhibited optimal in vitro tolerance in human corneal and conjunctival epithelial cells. No signs of ocular alteration or discomfort were observed when LAT-HA-LIP was administered to rabbits. Hypotensive studies revealed that, after a single eye drop, the effect of LAT-HA-LIP lasted 24 h longer than that of a marketed formulation and that relative ocular bioavailability was almost three times higher (*p* < 0.001).

As VEGF in choroidal neovascularization is a major cause of visual loss in the elderly and diabetics, gene therapy may offer an alternative treatment. However, siRNA instability and inefficient delivery are the main hindrances. To solve this problem, the developed trimethyl chitosan/HA nano-polyplexes loaded with a nano-sized siRNA had a narrow size distribution, no significant cytotoxicity, and proper cellular uptake. A remarkable gene silencing in HUVEC cells was shown. The intravitreally administered nano-polyplexes in rabbits overcame both the vitreous and retina barriers and reached the posterior tissues efficiently. Intravitreal injections of the vascular endothelial growth factor (VEGFR-2) siRNA nano-polyplexes significantly reduced the size of the laser-induced choroidal neovascularization, compared to the control group [141].

## 7. Treatment of Osteoarthritis and Rheumatoid Arthritis

Articular cartilage is most commonly damaged at the junction between bones and is progressively worsened by movement. The loss of the normal cartilage tissue can lead to the development of OA [28], which, from the clinical perspective, leads to joint pain and loss of function, and it is the leading cause of progressive disability [142]. Several challenges to the successful development of a functional OA graft are as follows: 1) catabolic enzymes in the joint that damage graft components of ECM, 2) adverse effects of the engineered scaffold material(s) on the biological potential of seeded cells, 3) limited mass transport in tissue-engineered grafts that prevent scaling up to clinically relevant sizes, and 4) differences in material and structural properties between healthy tissue and engineered grafts, which results in graft failure [143].

Hyaluronan, a component of the ECM of articular cartilage, can be loosely bound to CD44, which is highly expressed in chondrocytes and synoviocytes in OA. Hyaluronan has important regulatory functions in cartilage, especially when precartilaginous tissues are transformed into cartilage [144]. The molecular weight of HA in synovial fluid is between 6 and 7 MDa [46]. Hyaluronan provides viscoelasticity for synovial fluid. For this reason, HA can serve as a shock absorber, thereby allowing for smooth joint movements. Hyaluronan with a molecular weight of several megaDaltons, synthesized by HA synthases, is extruded into the SF by type B synoviocytes embedded within the synovium [145,146]. In synovial joints HA plays an important role in cell adhesion, morphogenesis, and the regulation of inflammation. However, in diseases such as OA and rheumatoid arthritis (RA), metabolism of HA and its distribution within the joint are altered. Lower HA levels can be attributed to an imbalance between HA synthesis and degradation linked with the increased activity of hyaluronidases. Consequently, the cartilage becomes vulnerable to wear and tear, resulting in its degradation, pain, stiffness, and functional impairment of synovial joints [144,147].

In the early stages of OA development, conservative nonsurgical management of OA and pharmacological therapies, such as the use of steroids and non-steroidal anti-inflammatory drugs (e.g., corticosteroids) or injections of HMW HA, can alleviate the OA symptoms [23,27,143,148,149,150]; however, they do not suppress the progression of OA. For patients with severe joint injuries or when the conservative treatment is not sufficient, surgical treatments such as joint replacement and osteotomy are recommended; however, long-term outcomes for patients can vary significantly.

One of the solutions can be to apply hydrogel scaffolds with high strength and resilience, which are appropriate in repairing or regenerating tissues and fast restoring the biomechanical function of tissues [23,27,143,150]. Specifically, HA scaffolds have been shown to have an impact even on the gene expression of chondrocytes, leading to higher expression of collagen type II, which in turn is representative of a regenerative cell phenotype, apart from suppressing genes associated with cartilage inflammation [144]. Stem cells, which have also been extensively studied in preclinical stages due to their anti-inflammatory, immunoregulatory, and regenerative properties, are expected to treat OA [23,27,143,150]. However, the environment of synovial joints is avascular for these cells upon injection [151,152]. This fact encouraged researchers to develop suitable injectable materials or systems for MSCs to enhance their function and survival and to promote cartilage protection, regeneration, and differentiation into chondrocytes. Hydrogels maximize the action of incorporated MSCs. Moreover, hydrogels have advantageous cartilage-like properties, since they contain collagen or HA moieties that interact with MSC receptors, thereby promoting cell adhesion [152].

Unlike OA, RA is a chronic inflammatory autoimmune disease that causes hyperplasia of synovial membranes, cartilage damage and bone destruction. Although the exact mechanism of RA remains unknown, activated macrophages in the inflamed joints play a crucial role in the progression of the disease via the production of pro-inflammatory cytokines such as TNF-α, IL-6, and IL-1.

For this reason, inhibiting the secretion of pro-inflammatory cytokines from activated macrophages has been the primary target of RA therapy. CD44 receptor is often overexpressed on the surface of activated macrophages in RA patients [153,154].

Despite significant advancements in the clinical treatment of RA, a major disadvantage is that long-term drug administration can lead to adaptive treatment tolerance, followed by reduced efficacy, the need for increased drug doses, and severe adverse effects. Currently, to overcome these problems, researchers have been developing nanotechnology-based nanomedicine for the treatment of RA. Multifunctional nanomedicine with targeted stimuli-responsive features has been one of the key ideas in designing more accessible formulations for efficient RA treatment. Such nanomedicine is able to postpone RA progression effectively due to its delivery and on-demand release of drugs at targeted sites in response to external or internal stimuli related to the RA pathophysiology without obvious adverse side effects on the normal tissues. Therefore, nanomedicine has gained interest from preclinical research scientists as well as clinical physicians worldwide [155].

**Figure 6:**
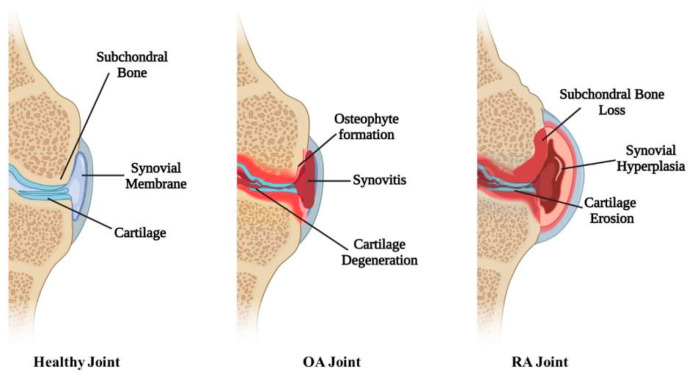
Healthy, OA and RA joints (adapted from Wen et al. [157]).

**Figure 7:**
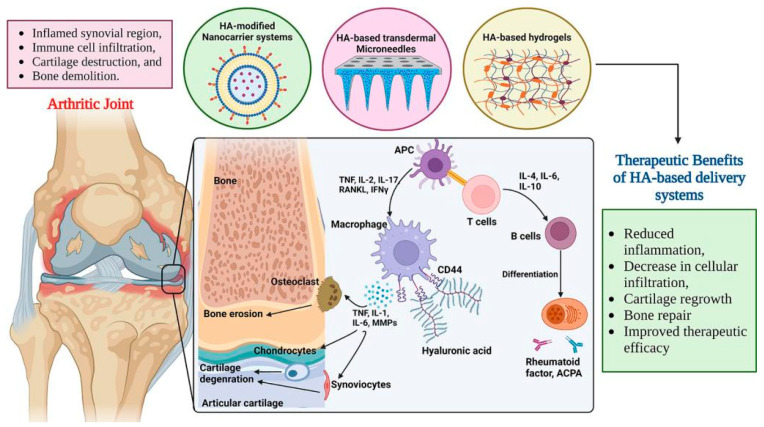
Treatment of RA using HA-modified nanocarriers, transdermal microneedles, and hydrogels. Reprinted with permission from Priya et al. [158]. Copyright 2025 Elsevier.

Hydrogels, as well as micro- and nanocarriers, proved to be very promising approaches to lower the number of injections to reduce the risk of infections and to prolong the residence time of drugs after intra-articular injection. Hyaluronic acid-, fibrin-, collagen-, and alginate-based hydrogels can be directly infused into defective sites without invasive surgical procedures due to their appropriate soft and biodegradable properties. With this regard, thermo-sensitive hydrogels became attractive in the past 20 years due to their simple injectability, high drug loading with sustained release, minor systemic side effects, and superior patient compliance [155,156]. Figure 6 depicts the healthy joint and differences between OA and RA joints, and Figure 7 illustrates the treatment of RA by using HA-based delivery systems.

To enhance the treatment of osteoarthritis, Greco et al. [159] synthesized HA/glycyl-l-histidyl-l-lysine/copper complexes with antioxidant, angiogenic, osteogenic, and synergic effects. The results highlighted copper’s role in promoting the expression and release of certain angiogenic, trophic, and osteogenic factors, including brain-derived neurotrophic factor, VEGF, as well as bone morphogenetic protein-2. Chen et al. [160] fabricated injectable HA hydrogel modified by aldehyde groups and AHAMA, which could enhance proliferation and migration of bone marrow stem cells (BMSCs). In a rat osteochondral defect model, the implanted hydrogel significantly promoted integration between neocartilage and host tissues and significantly improved cartilage regeneration. Hou et al. [28] fabricated structurally stable chondrocytes and HA-graft-amphiphilic gelatin microcapsules to serve as a biomimetic chondrocyte ECM environment. Cartilage tissue-specific gene expressions of collagen type II and SOX9 were upregulated in the presence of HA-graft-amphiphilic gelatin microcapsules in the early stage. Nabizadeh [161] examined for the first time a dual effect of chitosan/fisetin/kartogenin NPs incorporated into the HA hydrogel, which suppressed inflammation and supported cartilage regeneration. Min et al. [162] prepared HA/chitosan-poly(dioxanone) complex NPs loaded with bone morphogenic protein-7 (BMP-7), which were incorporated into alginate-poloxamer (ALG-POL)/silk hydrogel for constructing composite gels to achieve controlled release of BMP-7. Unlike the blank (ALG-POL/SF gels), synovium-derived mesenchymal stem cells seeded in the composite gel significantly expressed cartilage-related genes once they were seeded in the BMP-7 composite gel for two weeks and differentiated toward chondrogenesis. The research by Sharma et al. [163] demonstrated how HA-oleic acid micelles mixed in chitosan gel can deliver aceclofenac through the skin to help treat OA. The novel gel showed physico-chemical properties and significantly reduced pain and inflammatory response. Moreover, radiological and histopathological conditions were improved in monoiodoacetate-induced OA in animals. Yao et al. [164] prepared a biomimetic injectable di-self-crosslinking blend hydrogel by combining thiolated HA, maleimided HA, and collagen type I, which improved resistance to degradation and chondrocyte adhesion and proliferation, especially for increasing levels of gene expression linked to hyaline cartilage formation and polyproteoglycan secretion. Yin et al. [165] performed microfluidic technology to fabricate HA-based hydrogel microparticles for encapsulating exosomes. The results suggested that miR-99-b-3p regulated the degradation of cartilage ECM by targeting ADAMTS4 (a disintegrin and metalloproteinase with thrombospondin motifs 4). The upregulation of miR-99-b-3p in exosomes derived from ScAT stem cells could result in comparable or even higher effectiveness than exosomes derived from IPFP stem cells for OA treatment. Chen et al. [166] regulated cartilage inflammation and degeneration associated with abnormal IL-1β mRNA expression in OA, while the oligonucleotides were combined with gold nanorods to create spherical nucleic acids (SNAs) that enhanced stability and cell internalization efficiency. Furthermore, the complementary oligonucleotides were grafted onto HA to obtain DNA-grafted HA for SNAs delivery. The in vitro and in vivo experiments indicated that this system down-regulated catabolic proteases and up-regulated anabolic components in cartilage over extended periods of time to safeguard the chondrocytes against degenerative changes and impede the continual advancement of OA. To improve bone regeneration and locally treat osteoporosis, Svarca et al. [167] prepared HA/strontium ranelate (HA/SrRan), HA/calcium phosphate (HA/CaP) NPs, and HA/CaP NPs/SrRan hydrogels. Vu et al. [168] prepared hydrogel composites comprising alginate, *N,O*-carboxymethyl chitosan, and aldehyde HA loaded with biphasic calcium phosphate, which facilitated bone regeneration in the full-thickness calvarial defect mouse model, promoted cell proliferation, and had compressive strength. To provide a safe, efficient RA treatment with few adverse effects in rats with adjuvant arthritis, Makled et al. [169] administered intra-articularly collagen thermosensitive polyloxamer 407 (PCO)/melatonin (MEL) hydrogels encapsulated in hyalurosomes (HA-coated liposomes). This novel three-component hydrogel greatly enhanced joint healing, cartilage repair, pannus production, and cell infiltration when compared to PCO/MEL and blank PCO hydrogels. In 2020 Storozhylova et al. [170] developed an easily injectable controlled HA-fibrin hydrogel containing dexamethasone-loaded nanocapsules. The rheological properties of the system allowed for satisfactory syringeability and provided desirable mechanical properties for intra-articular application. The HA-fibrin hydrogels containing 30% (*v*/*v*) nanocapsules showed the capacity to control the release of dexamethasone for 72 h in simulated synovial fluid. The preliminary in vivo results using a galectin-3 inhibitor in an acute synovitis rat model showed a significant suppression of inflammation after intraarticular administration compared with the non-treated rats. Zewail et al. [171] fabricated hyalurosomes co-encapsulated with dexamethasone and luteolin. In a rat model of RA, 2.9-, 3.2-, 2.5-, and 2.7-fold decreases in matrix metalloproteinase 3 (MMP3), TNF-α, malondialdehyde, and IL1, respectively, were shown in comparison to the positive control group.

In gene therapy, Wang et al. [172] used PBA-modified HA hydrogel to encapsulate the siRNA-Fe_3_O_4_ NPs, which exhibited excellent biocompatibility, anti-inflammatory effects, and prolonged stable retention time in the knee joint. The hydrogel significantly attenuated cartilage degradation, synovitis, osteophyte formation, and subchondral bone sclerosis, as well as markedly improved physical activity and reduced pain in mice with the destabilized medial meniscus-induced OA. Zhao et al. [173] developed composite microspheres loaded with HA/chitosan siRNA NPs, which provided a sustained release of NPs, protected siRNA against nuclease degradation in the serum, and could readily cross the cellular membrane. They had greater pharmacodynamic effects than common microspheres and reduced the frequency of drug administration.

Table 1 summarizes HA-based systems with encapsulated drugs or antioxidants, targeted therapy, particle size, and period of release. Table 2 summarizes various HA-containing formulations, encapsulated cell types, matrix morphologies, therapeutic targets, and properties.

## 8. Future Directions

**Figure 8:**
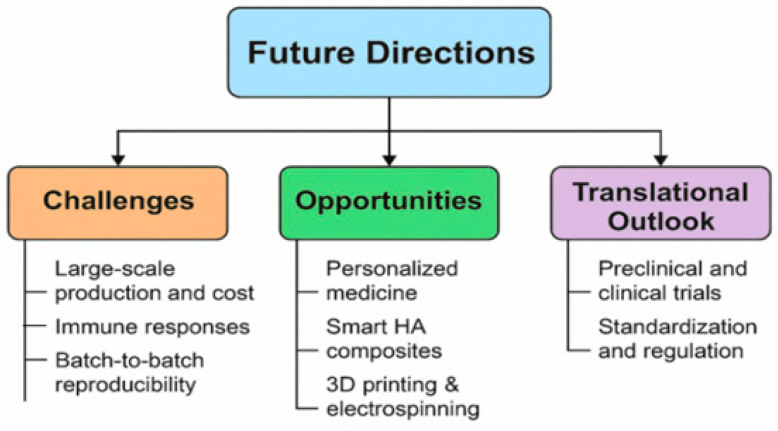
The summary of the future directions of HA-based encapsulation systems. The scheme highlights key challenges, emerging opportunities, and the translational outlook required to bridge laboratory-scale innovations with clinical applications.

Despite significant progress in the development of HA-based encapsulation systems, several obstacles remain to be solved before these technologies can be fully translated into clinical practice. As illustrated in Figure 8, one of the primary obstacles is the large-scale production and cost-effectiveness of advanced HA composites, particularly those involving NPs and multifunctional hydrogels. In addition, batch-to-batch reproducibility, material stability, and storage limitations continue to hinder consistency in manufacturing. Another key barrier is the potential for immune responses triggered by chemically modified HA or hybrid nanocarriers, which raises concerns regarding long-term therapeutic use.

Conversely, important opportunities are emerging that may accelerate clinical adoption. Advances in personalized medicine demonstrate the potential of tailoring HA formulations to patient-specific needs, especially for chronic conditions such as osteoarthritis, diabetes, and wound healing. Moreover, the development of smart, stimuli-responsive HA composites that can react to physiological cues (e.g., pH, enzymes, ROS) enables precise and controlled drug release. The integration of 3D printing and electrospinning technologies further opens possibilities for designing customizable scaffolds, advanced drug delivery systems, and multifunctional wound dressings with enhanced biological performance.

The translational outlook, as highlighted in Figure 8, underscores the urgent need to bridge the gap between laboratory innovation and clinical application. This requires rigorous preclinical and clinical trials to validate safety, efficacy, and long-term outcomes of HA-based encapsulation systems. Equally important is the standardization of protocols and regulatory frameworks, which will support approval processes and facilitate commercialization. By addressing these challenges while leveraging the outlined opportunities, HA-based encapsulation technologies hold strong potential to advance next-generation biomaterials and nanomedicines for regenerative medicine, targeted drug delivery, and tissue engineering.

## 9. Conclusions

Recently, hyaluronan has emerged as a versatile and valuable biopolymer in nano- and microencapsulation techniques for targeted drug delivery. Its unique physicochemical and biological properties, especially its biocompatibility, molecular weight, and the ability to form hydrogels or nanoparticles, make it an ideal candidate for developing advanced delivery systems with improved therapeutic efficacy, reduced side effects, and enhanced site-specific targeting. The combination of hyaluronan with other polymers, drugs, antioxidants, and nanomaterials has yielded promising outcomes in treating especially chronic diseases such as skin wounds, osteoarthritis, rheumatoid arthritis, diabetes, and ocular diseases. Moreover, recent research investigations into the hyaluronan-based encapsulation systems focus on the new opportunities for stem cell delivery and gene therapy and the development of pH- and ROS-responsive dual-layer hydrogels. The potential of nanoencapsulations is to develop smart/stimuli-responsive hydrogels and multifunctional composite nanoparticles to mimic biological systems by being coated with natural cell membranes or other biological components, which enhances their biocompatibility, targeting ability, immune evasion, and personalized medicine.

## Figures and Tables

**Table 1:** Hyaluronan-based encapsulation systems in therapeutic applications.

Components	Encapsulated Drug	Matrix Morphology	Targeted Therapy	Effects	Particle Size [nm]	Period of Drug Release	Ref.
HA	Curcumin and quercetin	NPs	Skin burns	MC3T3-E1 cell proliferation, improved penetration of curcumin and quercetin through the stratum corneum, 98% wound healing on day 28, granulation tissue was formed.	177 ± 11	72.4% curcumin and 87.7% quercetin in 70 h	[174]
HA/cyclodextrin (CD)	Paeonol (PAE, a natural substance)	Topical delivery carrier	Atopic dermatitis	The PAE retention rates of the HACD-PAE group in the stratum corneum and dermis were 3.35 and 1.78 times, respectively, higher than those of the PAE group. HACD could increase the gap of keratinocytes by interacting with corneum lipids and loosening the keratin, with high efficacy on atopic dermatitis mice.	177 ± 9.19	40% in 12 h	[175]
HA/polycaprolactone-b-PEG-b-polycaprolactone	Curcumin	Hydrogel	Skin wound	Enhanced angiogenesis, the formation of collagen fibers.	129	72% in 12 h	[176]
Octadecylamine-modified HA	Curcumin	Micelles	Skin burns	Significantly increased skin penetration and retention of curcumin, higher analgesic and anti-inflammatory activities in vivo when compared with curcumin solution. Curcumin’s transdermal penetration mechanism may be associated with HA’s hydration of the stratum corneum.	165.64	48 h at 4 °C in 21 days	[177]
HA/bilosomes	Au-triptolide	Hydrogel	RA	Excellent cellular uptake and targeted delivery efficiency for triptolide, elongation of circulatory residence time, enhancement of intraarticular bioavailability, and higher in vivo antiarthritic efficacy compared to uncoated triptolide/bilosomes.	164.2	60% release with near-infrared radiation (NIR), 30% release without NIR in 24 h.	[178]
HA	Teriflunomide	Lipid carriers	RA	High stability, superior cytotoxicity and binding affinity to CD44 receptors compared with teriflunomide itself, increased teriflunomide bioavailability, reduced TNF-α serum levels, and improved joint healing.	284.9 ± 3.8	100% in 30 h	[179]
Lipid carriers coated with chondroitin sulfate, HA, or chitosan	Leflunomide	Hydrogel	OA	Fastest recovery of rats, improved cartilage thickness, chondrocyte proliferation and neovascularization, reduced TNF-α level 4–5-fold relative to positive control, limited chondrocyte apoptosis, and production of pro-inflammatory cytokines.	101.5−153.8	100% in 44 h	[156]
HA/oleic acid	Aceclofenac	Micelles	OA	A significant reduction in pain and inflammation and improved radiological and histopathological conditions in animals.	245 ± 7.68	80% in 50 h	[163]
HA	Celecoxib	Nanocapsules	OA	Higher efficacy of celecoxib nanocapsules compared to celecoxib suspension in a monoiodoacetate-induced OA rat model.	254.9 ± 3.06	100% in 7 days	[180]
HA/gelatin	Kaempferol	NPs	OA	Significant reduction in subchondral sclerosis and the severity of OA in the ACLT rat model, attenuated inflammation and ECM degradation, and restored cartilage thickness.	88.62 ± 3.90	18% over 48 h	[181]
HA/poloxamer	Ketoprofen-loaded transethosomes	Hydrogel	OA	The X-ray imaging of the treated group showed intact meniscus, healthy articular joints, and the same normal synovial lining as in the healthy control group, reduced pain and inflammation.	110.0 ± 1.70	Approx. 90% in 80 h	[182]
PLGA NPs/HA	Bovine serum albumin	Hydrogel	Ocular neovascular diseases − AMD	Retained 75% of its wet weight without losing its integrity, and the release of the model drug at the rate of 0.4 g/day for more than 2 months under physiological conditions improved bioavailability of the drug by penetrating deep into the retinal layers.	54.81 ± 7.95	Cumulative rapid release within 24 h, followed by a linear release lasting up to 56 days.	[183]
Chitosan/sulfobutylether-β-CD/thiolated HA	Indomethacin	NPs	Ophthalmology (anterior segment inflammation diseases)	Increased residential time in the conjunctival sac, no irritation or toxicity. In contrast, the uncoated NPs displayed better permeating properties since they are smaller and could be further exploited for the treatment of posterior segment diseases.	340 ± 7	80% in 6 h	[184]
Chitosan/HA	Erythropoietin	NPs	Ophthalmology	More rapid permeation through porcine conjunctiva, followed by sclera and cornea, and noncytotoxicity on ARPE-19 and HaCaT cell lines enhanced its retention time and permeation through the different ocular membranes.	≤300	80% release of erythropoetin from simulated tear fluid in 6 h	[185]
*N,N*-dodecyl, methyl-polyethylenimine/HA	Vancomycin	NPs	Ophthalmology (bacterial endophthalmitis)	Nontoxic to ARPE-19 cells, non-irritating to the chorioallantoic membrane, and no changes in retinal functions.	154 ± 3	58% over 96 h	[186]
Zein and HA	Ciprofloxacin	NPs	Ophthalmology (bacterial conjunctivitis)	A possible alternative to the current antibacterial topical dosage forms available on the market for treating.	200	Approx. 100% within 24 h	[187]
HA/chitosan	Curcumin liposomes, resveratrol	Hydrogel	Diabetic retinopathy	The successful integration of liposomes and hydrogels in the creation of 3D-printed hydrogel scaffolds enabled the delivery of resveratrol and curcumin. Microfluidics and 3D bioprinting can be effectively combined to produce versatile carriers capable of accommodating various active pharmaceutical ingredients.	<200	75% of resveratrol and 10% of curcumin in 24 h	[188]

**Table 2:** Overview of HA-based materials and composites used for cell encapsulation and tissue engineering applications.

Components/Coating Material	Encapsulated Cells	Matrix Morphology	Targeted Therapy	Effects, Properties	Ref.
Collagen/tyramine/HA	Amniotic mesenchymal stem cell metabolite products	Hydrogel	Skin wounds	Highly resistant against enzymatic degradation, with a high degree of hydration and cell viability, collagen improved cell attachment and survival.	[189]
Agarose-collagen type I/dermatan sulfate, HA, elastin	NIH-3T3 cells	Hydrogel	Skin wounds	High cytocompatibility and hemocompatibility, supported cell growth and metabolic activity, created 3D mesh structures with potential clinical application as a cellular skin substitute.	[190]
HA/dopamine	BMSCs and growth factors	Hydrogel	Skin wounds	Significantly accelerated healing of acute full-thickness skin wounds, resulting in the formation of appendages such as hair follicles and minimal scarring.	[191]
HA-CD and HA-adamantane	Human corneal epithelial cells	Hydrogel	Opthalmology	Absorbed within the corneal stroma over time, modulated mesenchymal corneal stromal cell secretome production, reduced cellularity and inflammation of the anterior stroma, and significantly mitigated corneal edema compared to treatment with linear HA and untreated control eyes.	[192]
Fibrin and thiolated HA	Primary human fibroblasts	Hydrogel	Skin wound healing	Reduction in contraction, more homogeneous keratin 10 (K10) expressions in the supra-basal layer of the epidermis; enhanced stratum corneum formation for the constructs containing HA.	[193]
HAMA/insulin-like growth factor 1 (IGF-1)	Keratinocytes	Hydrogel	Skin wound healing	HAMA (3% w/v) hydrogel was the most appropriate for the 3D cell culture. Incorporating IGF-1 into the hydrogel in a dose-dependent manner significantly enhanced the viability of the encapsulated keratinocytes. The hydrogels were shown to be cytocompatible. The keratinocytes were shown to grow in 3D fashion.	[194]
F127 diacrylate/HA	NIH-3T3 cells	Micelles	Tissue repair	Better physical–chemical properties, using a 3D printer led to precise structures with high cell viability. The viscoelastic microenvironment fosters fibroblast spreading within the bioprinted matrices and supports the development of a biomimetic skin construct characterized by multilayer keratinocytes on the surface. The healing was accelerated by inflammation suppression, angiogenesis, and ECM promotion using a full-thickness mouse skin wound model.	[195]
PEGDA and HAMA	Canine islets	Hydrogel microspheres	Diabetes mellitus type 1	In diabetic NOD mice, PEGDA microspheres reversed diabetes for the length of the study (up to 16 weeks). On the contrary, islets encapsulated in HAMA microspheres restored normoglycemia, but only transiently (3–4 weeks). Transplanted nonencapsulated canine islets did not restore normoglycemia for any length of time.	[196]
HA of 0.1 and 1.2 MDa crosslinked with bis(β-isocyanatoethyl) disulfide	Pancreatic beta cells from the MIN-6 lineage	Hydrogel	Diabetes mellitus type I	Gels (0.1 MDa HA) had higher crosslinking densities and consequently, higher tensile and storage loss moduli. Both HMW and LMW HAs were biocompatible. Gels maintained cell viability, and they did not activate the immune system. Due to sex dimorphism and hormonal variances, female mice were shown to be more resistant to the inducing effects of streptozotocin, where hyperglycemia was achieved in 48% of the cohort. Moreover, single-cell encapsulation did not revert hyperglycaemia after transplantation due to the lack of cell–cell interactions.	[197]
HA/alginate	MSC spheroids	Microcapsules	Stem cell-based therapies	Enhanced secretion of various growth factors was found from MSC spheroids, a significant promotion of angiogenesis by MSC spheroids compared to the controls (i.e., MSCs and MSC spheroids), which is likely because of the higher retention of MSC spheroid forms in the microcapsules.	[198]
HA	BMSCs-conditioned media	Hydrogel	OA	Enhanced beneficial effect of HA in treating degenerative changes in articulating surfaces associated with arthritic temporomandibular joints in rats, reduced toxicity and side effects, increased bioavailability, and minimized off-target activity.	[199]
HA/chitosan coacervate	Rat BMSCs	Hydrogel	Cartilage repair	Chondrogenic induction of encapsulated BMSCs within coacervate demonstrated remarkable cellular viability in addition to the elevated expression levels of chondrogenic markers such as sex-determining region Y-box 9 protein, aggrecan, cartilage oligomeric matrix protein, and collagen type II.	[151]
HA/hydroxyapatite	L929 fibroblasts	Hydrogel	Bone repair	Excellent cytocompatibility and supported adhesion and proliferation of cells under 3D culture conditions.	[200]
Enzymatically crosslinked HA	Human auricular chondrocytes	Microgel bioink	Cartilage regeneration	Excellent rheological properties, the granular hydrogels supported the homogeneous development of mature cartilage-like tissues in vitro. After 6 weeks of in vivo implantation, small-diameter microgels formed stable constructs with low immunogenicity and continuous tissue maturation. Conversely, increasing the microgel size resulted in an increased inflammatory response, with limited stability in vivo.	[201]
HA/tyramine/silk-fibroin	Articular chondrocytes	Hydrogel	Cartilage repair	Cytocompatible, promoted the expression of cartilage matrix proteins, while the most prominent chondrogenic effects were observed in hydrogels with HA: silk fibroin in the polymeric ratio 20:80. Among the hydrogels loaded with anabolic and anti-inflammatory drugs, the HA20/SF80 hydrogel demonstrated the longest and most sustained release profile over time, which is desirable for the extended treatment duration typically required for OA joints.	[202]
HAMA, collagen type I, and chitosan	BMSCs or primary articular chondrocytes	Hydrogel	Cartilage repair	Chondrocytes exhibited superior growth and matrix deposition compared to either chondrogenically induced BMSCs or a mixed polyelectrolyte complex microcapsule culture containing both chondrocytes and BMSCs.	[143]

## Abbreviations

The following abbreviations are used in this manuscript:

ALG-POL	Alginate-poloxamer
AMD	Age-related macular degeneration
AHAMA	Anhydride and methacrylic-modified hyaluronan hydrogel
BMP-7	Bone morphogenic protein 7
BSA	Bovine serum albumin
CD	Cyclodextrin
DES	Dry eye syndrome
DFO	Deferoxamine
DR	Diabetic retinopathy
DSP	Dexamethasone sodium phosphate
ECM	Extracellular matrix
Exos	Exosome
GelMA	Methacrylated gelatin
GOx	Glucose oxidase
HA	Hyaluronan
HAMA	Hyaluronan methacrylate
HMW	High molecular weight
HUVEC	Human umbilical vein endothelial cell
IGF-1	Insulin-like growth factor
IL	Interleukin
LAT-HA-LIP	Latanoprost-loaded phosphatidylcholine liposomes with hyaluronan
LMW	Low molecular weight
MEL	Melatonin
MMP	Metalloproteinase
MSc	Mesenchymal stem cells
NF-κB	Nuclear factor kappa B
NH	Nanohydrogel
NIR	Near-infrared radiation
NPs	Nanoparticles
OA	Osteoarthritis
ODDs	Ocular drug delivery systems
PAE	Paeonol
PBA	Phenylboronic acid
PEG	Polyethylene glycol
PCO	Collagen thermo-sensitive poloxamer
PEGDA	Poly(ethylene glycol)diacrylate
PLGA	Poly(lactic-co-glycolid acid)
PVA	Polyvinylalcohol
RA	Rheumatoid arthritis
RGD	Arginylglycylaspartic acid
ROS	Reactive oxygen species
RNV	Retinal neovascularization
SCN	Curcumin/silk/HA
SH	Silk/HA
siRNA	Silent interfering RNA
SNAs	Spherical nucleic acid
SrRan	Strontium ranelate
TLR-4	Toll-like receptor
TNF-α	Tumor necrosis factor α
VEGF	Vascular endothelial growth factor
